# Supplementary material for: The genome sequence of the Obscure Wainscot moth, Leucania obsoleta (Hubner, 1803) (Lepidoptera: Noctuidae)
Source: Wellcome Open Res. 2026 Sep 14;11:660. [Version 1] doi: 10.12688/wellcomeopenres.27815.1 (PMC13720295; doi:10.12688/wellcomeopenres.27815.1)
Supplement: Peer review history

# Reviewer report: Reviewer response for version 1

**DOI:** 10.21956/wellcomeopenres.30608.r175622

**Published:** 2026-10-29

**Authors:** Bengt Hansson

**Affiliations:** 1 Lund University, Lund, Sweden

This article presents a high-quality genome assembly for the Obscure Wainscot moth, *Leucania obsoleta*, generated as part of Project Psyche. The authors provide assemblies of both haplotypes, with total lengths of approx. 700 Mb. The haplotype 1 assembly is highly contiguous, with almost 100% of the sequence assigned to 31 chromosomal pseudomolecules, including the Z chromosome. The mitochondrial genome has also been assembled.

 Overall, I find this to be a robust and valuable genome assembly note. The assembly is of high quality and represents a useful genomic resource for *L. obsoleta* and for future comparative genomic studies of Lepidoptera. The presentation is clear, and the scope of the manuscript is appropriate for a genome resource article.

 I answered “yes” to all of the assessment questions and did not identify any scientific issues that, in my view, require correction before the article can be considered scientifically sound. In particular, I found no major concerns regarding the assembly, its presentation, or the conclusions drawn from the work.

 I therefore have no essential revisions to request and consider the article scientifically sound in its present form.

Are the datasets clearly presented in a usable and accessible format, and the assembly and annotation available in an appropriate subject-specific repository?

Yes

Are sufficient details of the sequencing and extraction, software used, and materials provided to allow replication by others?

Yes

Are the rationale for sequencing the genome and the species significance clearly described?

Yes

Are the protocols appropriate and is the work technically sound?

Yes

Reviewer Expertise:

Evolutionary biology, population genetics, comparative genomics

I confirm that I have read this submission and believe that I have an appropriate level of expertise to confirm that it is of an acceptable scientific standard.

# Reviewer report: Reviewer response for version 1

**Published:** 2026-09-14

**Authors:** Automated Benchmarking

**Affiliations:** 1 Wellcome Sanger Institute, Hinxton, UK

As this article is a Genome Note, Automated Benchmarking has been used to determine whether the reported assembly passes three defined checks established by the Earth BioGenome Project: the raw and assembled data are publicly available, the Earth BioGenome Project three-part metric is met, and the BUSCO score passes the stated threshold. For Leucania obsoleta (ilLeuObso1), all three checks passed. Data available: pass. ENA study PRJEB87475, raw-read accessions ERR14835542 and ERR14782889, and assembly accessions GCA_965615905.1 and GCA_965615885.1 are public. Earth BioGenome Project three-part metric: pass. Haplotype 1 is reported as 7.C.Q67, meeting the 6.C.Q40 reference standard. BUSCO: pass. Haplotype 1, which is proposed as the reference assembly for this species, has 99.5% complete BUSCOs (98.9% single-copy and 0.6% duplicated). BUSCO software version: 5.8.3; lineage dataset: lepidoptera_odb10. It meets the target of greater than 90% complete and single-copy BUSCOs. This decision uses values reported in the Genome Note and public accession records.

Reviewer Expertise:

NA

I confirm that I have read this submission and believe that I have an appropriate level of expertise to confirm that it is of an acceptable scientific standard.
